# Influences of *APOA5* Variants on Plasma Triglyceride Levels in Uyghur Population

**DOI:** 10.1371/journal.pone.0110258

**Published:** 2014-10-14

**Authors:** Shuyuan Li, Bin Hu, Yi Wang, Di Wu, Li Jin, Xiaofeng Wang

**Affiliations:** 1 Ministry of Education Key Laboratory of Contemporary Anthropology and State Key Laboratory of Genetic Engineering, Collaborative Innovation Center for Genetics and Development, School of Life Sciences, Fudan University, Shanghai, China; 2 Fudan-Taizhou Institute of Health Sciences, Taizhou, Jiangsu, China; Cincinnati Children's Hospital Medical Center, United States of America

## Abstract

**Objective:**

Single nucleotide polymorphisms (SNPs) in apolipoprotein A5 (*APOA5*) gene are associated with triglyceride (TG) levels. However, the minor allele frequencies and linkage disequilibriums (LDs) of the SNPs in addition to their effects on TG levels vary greatly between Caucasians and East Asians. The distributions of the SNPs/haplotypes and their associations with TG levels in Uyghur population, an admixture population of Caucasians and East Asians, have not been reported to date. Here, we performed a cross-sectional study to address these.

**Methods:**

Genotyping of four SNPs in *APOA5* (rs662799, rs3135506, rs2075291, and rs2266788) was performed in 1174 unrelated Uyghur subjects. SNP/haplotype and TG association analyses were conducted.

**Results:**

The frequencies of the SNPs in Uyghurs were in between those in Caucasians and East Asians. The LD between rs662799 and rs2266788 in Uyghurs was stronger than that in East Asians but weaker than that in Caucasians, and the four SNPs resulted in four haplotypes (TGGT, CGGC, TCGT, and CGTT arranged in the order of rs662799, rs3135506, rs2075291, and rs2266788) representing 99.2% of the population. All the four SNPs were significantly associated with TG levels. Compared with non-carriers, carriers of rs662799-C, rs3135506-C, rs2075291-T, and rs2266788-C alleles had 16.0%, 15.1%, 17.1%, and 12.4% higher TG levels, respectively. When haplotype TGGT was defined as the reference, the haplotypes CGGC, TCGT, and CGTT resulted in 16.1%, 19.0%, and 19.8% higher TG levels, respectively. The proportions of variance in TG explained by *APOA5* locus were 2.5%, 0.3%, 0.4%, and 1.9% for single SNP rs662799, rs3135506, rs2075291, and rs2266788, respectively, and 3.0% for the haplotypes constructed by them.

**Conclusions:**

The association profiles between the SNPs and haplotypes at *APOA5* locus and TG levels in this admixture population differed from those in Caucasians and East Asians. The functions of these SNPs and haplotypes need to be elucidated comprehensively.

## Introduction

The *APOA5* gene is located approximately 27 kb downstream from the APOA1/C3/A4 gene cluster on human chromosome 11q23 [1], [2]. The protein product of the *APOA5* gene, apoA-V, is a component of several lipoprotein fractions, including very low-density lipoprotein (VLDL), high-density lipoprotein, and chylomicrons [3]. Although the exact function of apoA-V is not yet clear, it was demonstrated to be able to reduce triglyceride (TG) levels by activating lipoprotein lipase, thus inhibiting VLDL-TG production and stimulating VLDL-TG hydrolysis [4].

Five single nucleotide polymorphisms (SNPs), including rs662799 (−1131 T>C), rs651821 (−3 A>G), rs3135506 (S19W, c.56C>G), rs2072560 (715 G>T, which was previously referred to as IVS3+476G>T), and rs2266788 (1891 T>C, c.158C>T, which was previously referred to as c.1259T>C), were initially identified to be associated with plasma TG levels in Caucasians [5]–[10]. Except for rs3135506, the other four SNPs were in almost complete linkage disequilibrium (LD), with *r* ^2^ ranging from 0.87 to 1, resulting in the three following haplotypes: haplotype APOA5*1, which is the wild-type haplotype as defined by the presence of major alleles at all of the sites; haplotype APOA5*2, which is defined by the rare alleles at four sites, including rs662799, rs651821, rs2072560, and rs2266788; and haplotype APOA5*3, which is distinguished from APOA5*1 by the rare allele of the SNP rs3135506 [5], [6]. Compared with the most common haplotype APOA5*1, the haplotypes APOA5*2 and APOA5*3 were also associated with higher TG levels in Caucasians [5]–[7]. However, further studies in East Asians revealed contrasting results, indicating that the SNP rs3135506 and haplotype APOA5*3 were rare and not associated with TG levels in this population [11]–[13]. The frequencies of the minor alleles of the five SNPs vary significantly in East Asians compared with those in Caucasians. The minor allele frequencies (MAFs) of the five SNPs were in the range of 0.06 to 0.08 in the Caucasians; however, the MAFs of rs662799, rs651821, rs2072560, and rs2266788 increased to 0.20 to 0.30, and the MAF of rs3135506 decreased to less than 0.01 in the East Asians [5], [6], [11]–[15]. The LD patterns of the five SNPs in East Asians also differed from those in Caucasians. Tight LDs between rs662799 and rs651821 (*r* ^2^≥0.99), and also between rs2072560 and rs2266788 (*r* ^2^≥0.9) were observed in East Asians, whereas the LDs between rs662799/rs651821 and rs2072560/rs2266788 were less prominent, with *r* ^2^ ranging from 0.6 to 0.7 in this population [13], [14]. The differing LD patterns also made the haplotype APOA5*4 defined by the common allele of rs662799/rs651821 with the rare allele of rs2072560/rs2266788, which was not detected in Caucasians, can be observed in East Asians. The haplotype APOA5*4 was also associated with familial combined hyperlipidemia [14]. To date, the mechanism of the SNPs or haplotypes that are associated with TG levels have not been elucidated and the causal SNPs remain to be identified. Moreover, another emerging SNP, rs2075291 (c.553 G>T, G185C), was also found to be associated with TG levels in East Asians only [15]–[19]. The combined effects of rs2075291 in addition to the aforementioned five SNPs on TG levels need to be studied comprehensively.

The Uyghur population, who reside in the Xinjiang Autonomous Region in China, represent a classic admixture population with a genetic background of Caucasians (40%) and East Asians (60%) [20], [21]. Because this population was recently admixed, the increased extent of LD between markers may facilitate the genomic mapping of complex disease genes. In addition, the population stratification is extremely low among Uyghurs in different areas of Xinjiang (*Fst* = 0.0009), minimizing potential confounding effects due to stratification in genetic association studies [21], [22]. Notably, Uyghurs show a higher prevalence of hypertriglyceridemia compared with the Hans and Kazaks, who also live in the same area [23], [24]. All of these advantages provide us with an opportunity to map the genetic variants of TG levels in this ethnic group. However, the genetic architectures that modulate TG levels in uyghurs remain unknown. Therefore, we performed a cross-sectional study to evaluate the genotype and haplotype frequencies of the SNPs in APOA5 and their associations with plasma TG levels in the Uyghur population.

## Methods

### Study subjects

Our study sample consisted of 1174 unrelated Uyghur subjects (404 men and 770 women with a mean age of 51.5±11.2 years) who were recruited from March to May of 2005 and April of 2006 from four villages in the Tulupan District of the Xinjiang Uyghur Autonomous Region. All of the participants were of Uyghur ancestry and had resided in Xinjiang for over 20 years. Subjects who reported receiving lipid-lowering medications for the treatment of dyslipidemia and who reported having tumors, autoimmune diseases, or hematological diseases were excluded. Written informed consent was obtained from each participant, and all of the protocols were approved by the Human Ethics Committee of Fudan University.

### Data collection

A standard questionnaire was administered by trained staff to obtain information on demographic characteristics, personal medical history, and lifestyle risk factors, etc. Ever smokers were those who had smoked regularly for at least 6 months. Alcohol consumption was defined as a dichotomous variable, and individuals who consumed more than 3 drinks per week were considered to be ever drinkers. All of the participants received a physical examination and blood tests at a local hospital after overnight fasting. A standardized mercury sphygmomanometer was used to measure systolic blood pressure (SBP) and diastolic blood pressure (DBP), and these measurements were obtained by two cardiologists. For the body weight and height measurements, the subjects wore only light indoor clothing and no shoes. BMI was calculated by dividing weight (kg) by height squared (m^2^). The waist circumference was measured midway between the caudal point of the costal arch, as palpated laterally, and the iliac crest. The blood specimens were drawn after overnight fasting, immediately subjected to centrifugation, and analyzed within 8 h for glucose (Glu), TG, and total cholesterol (TC). The clinical characteristics of the studied population are summarized in Table 1.

**Table 1:** Clinical characteristics of the studied subjects.

	Total
N	1174
Age (year)	51.5±11.2
Men (%)	404 (34.5)
BMI (kg/m^2^)$	26.6±4.57
Waist (cm)	88.3±11.4
Ever smoker (%)	163 (14.7)
Ever drinker (%)	63 (5.8)
SBP (mm Hg)	137.6±27.7
DBP (mm Hg)	83.8±15.0
TC (mmol/L)	4.54±1.04
TG (mmol/L)	1.56±1.10
Glu (mmol/L)	5.74±1.78

Values shown are numbers (frequencies) for categorical variables and mean ± standard deviation for continuous variables.

$ calculated by dividing weight (kg) by height squared (m^2^).

### DNA extraction, SNP selection and genotyping

Genomic DNA was extracted from EDTA-anticoagulated peripheral blood using a standard method. As previously mentioned, there were 6 SNPs, including rs662799, rs651821, rs3135506, rs2072560, rs2266788, and rs2075291, that were reported to be associated with TG levels in Caucasians or East Asians. As rs662799 and rs651821, as well as rs2072560 and rs2266788, were in strong LD in both Caucasians and East Asians, indicating they may be representative of each other. We only selected two SNPs, rs662799 and rs2266788, from the four SNPs in an effort to be cost efficient. The genotyping of the four SNPs in *APOA5*, including rs662799, rs3135506, rs2075291, and rs2266788, was performed using the TaqMan SNP Genotyping Assay (Applied Biosystems, Foster City, CA, USA). All of the genotyping success rates for these four loci were >99%. To assess reproducibility, 1% of the samples were analyzed in duplicate, and their genotypes were found to be 100% concordant.

### Statistical analysis

Deviations from Hardy–Weinberg equilibrium for the genetic variants were tested by a chi-squared test. Pair-wise D’and *r* ^2^ for all of the studied SNPs were calculated, and linkage disequilibrium structures were constructed by the Haploview software [25]. The haplotypes and their frequencies were inferred using the PHASE version 2.1.1 software (www.stat.washington.edu/stephens/phase/download.html) and haplotype-based association analysis was conducted based on the inferred haplotype data. For the function prediction of the SNP rs2075291, two *in silico* tools (PolyPhen and SIFT) were applied using the SeattleSeq Genomic Variation Server (http://snp.gs.washington.edu/SeattleSeqAnnotation134/). All of the other data analyses were performed with the SAS statistical software (release 9.1; SAS Institute Inc, Cary, NC, USA). The plasma TG levels were log-transformed to obtain approximate normal distributions for performing the statistical tests. A linear regression analysis was performed to identify any associations between the SNPs, haplotypes and plasma TG levels and the analysis was performed either without or with adjustments for confounding risk factors in the regression model. The variances that were explained by the *APOA5* SNPs or haplotypes were estimated by subtracting the total coefficient of determination (R^2^) based on the regression model without the *APOA5* genotype from that of the same model incorporating the *APOA5* genotype and adjusting for age, gender, BMI, smoking, and drinking. Because the minor allele frequency of rs3135506 was low, we combined the heterozygotes and homozygotes of the minor allele to increase the statistical power (dominant genetic model). For the other SNPs, an additive genetic model of the minor allele was assumed. For the SNP association analysis, a pre-specified threshold of *P*<0.0125 was used for the multiple correction significance (corresponding to *P*<0.05 after adjusting for four loci) while *P*<0.05 was considered to be significant in the other analyses.

To compare the frequencies of the minor allele of SNPs and haplotypes in Uyghurs with those in Caucasians and East Asians, the frequencies of the studied SNPs in the NCBI and Hapmap databases were collected and the PubMed, HugeNavigator, and Science Citation Index (ISI Web of Science) databases were searched to collect all of the publications involving the *APOA5* (last search update: 30th March 2014). The following key words were used: ‘‘apolipoprotein A5’’, OR “*APOA5*”, in combination with “polymorphis*”, OR “SNP*”, OR ‘‘single nucleotide’’, OR “variant*”, OR “genotype*”, OR “mutation*”. The references lists were further retrieved from identified publications to avoid missing any relevant articles. Only the studies met the following criteria were included: (1) the study were conducted in Caucasians or East Asians; (2) the three genotypes of rs662799, rs3135506, rs2075291, or rs2266788 were reported or the MAF of the SNPs and the frequencies of the haplotypes were available; (3) the genotype distributions should meet the Hardy–Weinberg equilibrium; (4) data were used from only apparently healthy individuals (ie, people selected from the hospital without known coronary or other diseases or from the general population). Animal studies, case reports, review articles, reports with incomplete data, and family studies were excluded. The genotype distributions of the SNPs and haplotypes were extracted and the MAF of the SNPs and the frequencies of the haplotypes were derived from weighted averages of the frequencies that were reported in the articles in addition to the NCBI and Hapmap databases.

## Results

### Frequencies of studied polymorphisms and haplotypes in uyghur population

**Figure 1:**
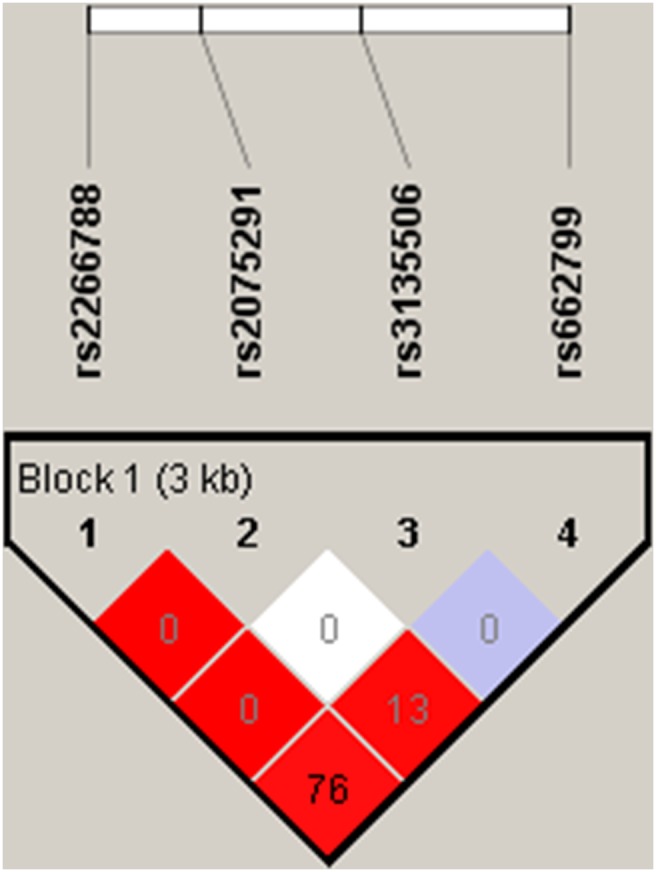
Haploview LD graph of the studied polymorphisms of APOA5 in general Uyghur population. Pairwise linkage disequilibrium is expressed as D’ (different colors) and *r* ^2^ (numbers).

The distributions of the genotypes of the four SNPs and the haplotypes that were derived from them are presented in Tables 2 and 3. The genotypes at all of the sites were consistent with Hardy-Weinberg equilibrium. The minor allele frequencies of rs662799, rs3135506, rs2075291, and rs2266788 were 0.19, 0.03, 0.03, and 0.16, respectively. The pairwise LD between the four SNPs in the total Uyghur population, which was expressed as D' and *r* ^2^, is presented in Figure 1. The LD between rs662799 and rs2266788 in Uyghurs was stronger than that in East Asians but weaker than that in Caucasians (*r* ^2^ = 0.75, *D*’ = 0.97). However, the LDs between the SNP rs3135506 or rs2075291 with the other three SNPs were weak with *r* ^2^ ranging from 0.002 to 0.13. We identified four major haplotypes as follows: TGGT (APOA5*1), CGGC (APOA5*2), TCGT (APOA5*3), and CGTT (APOA5*4, the four SNPs were ordered from 5′ to 3′) with frequencies of greater than 0.01. The most common haplotype, TGGT, represented 77.1% of all of the haplotypes; the other three haplotypes, CGGC, TCGT, and CGTT, accounted for 15.4%, 3.5%, and 3.2%, respectively. In total, these four haplotypes accounted for 99.2% of all of the haplotypes in this population.

**Table 2:** Association between the SNPs in *APOA5* and plasma TG levels in the general Uyghur population.

SNPs	Genotype	N (%)	TG	*P* *	*P* #
rs662799	TT	762 (65.4)	1.46±0.93	3.99×10^−7^	4.38×10^−8^
	CT	363 (31.1)	1.69±1.25		
	CC	41 (3.5)	2.37±2.03		
rs3135506	GG	1091 (93.3)	1.54±1.07	0.027	0.041
	GC/CC	78 (6.7)	1.82±1.46		
rs2075291	GG	1089 (93.7)	1.53±1.05	0.017	0.027
	GT	73 (6.3)	1.94±1.68		
rs2266788	TT	827 (71.2)	1.51±1.02	7.01×10^−5^	3.24×10^−6^
	CT	299 (25.8)	1.63±1.17		
	CC	35 (3.0)	2.41±2.01		

*unadjusted *P* value.

# adjusted for age, gender, BMI, smoking, and drinking.

Bold fonts represent significant difference after multiple correction (*P*<0.0125).

**Table 3:** Association between the haplotypes in *APOA5* and plasma TG levels in the general Uyghur population.

Haplotypes$	Freq (%)	TG	*P* *	*P* #
TGGT	77.1	1.49±0.98		
CGGC	15.4	1.79±1.42	3.17×10^−6^	2.35×10^−7^
TCGT	3.5	1.84±1.48	0.005	0.011
CGTT	3.2	1.94±1.70	0.006	0.010

$ The four SNPs in the haplotypes were arranged in the order of 5′ to 3′, i.e., rs662799, rs3135506, rs2075291, and rs2266788.

*unadjusted *P* value.

# adjusted for age, gender, BMI, smoking, and drinking.

Bold fonts represent significant difference after multiple correction (*P*<0.0125).

**Figure 2:**
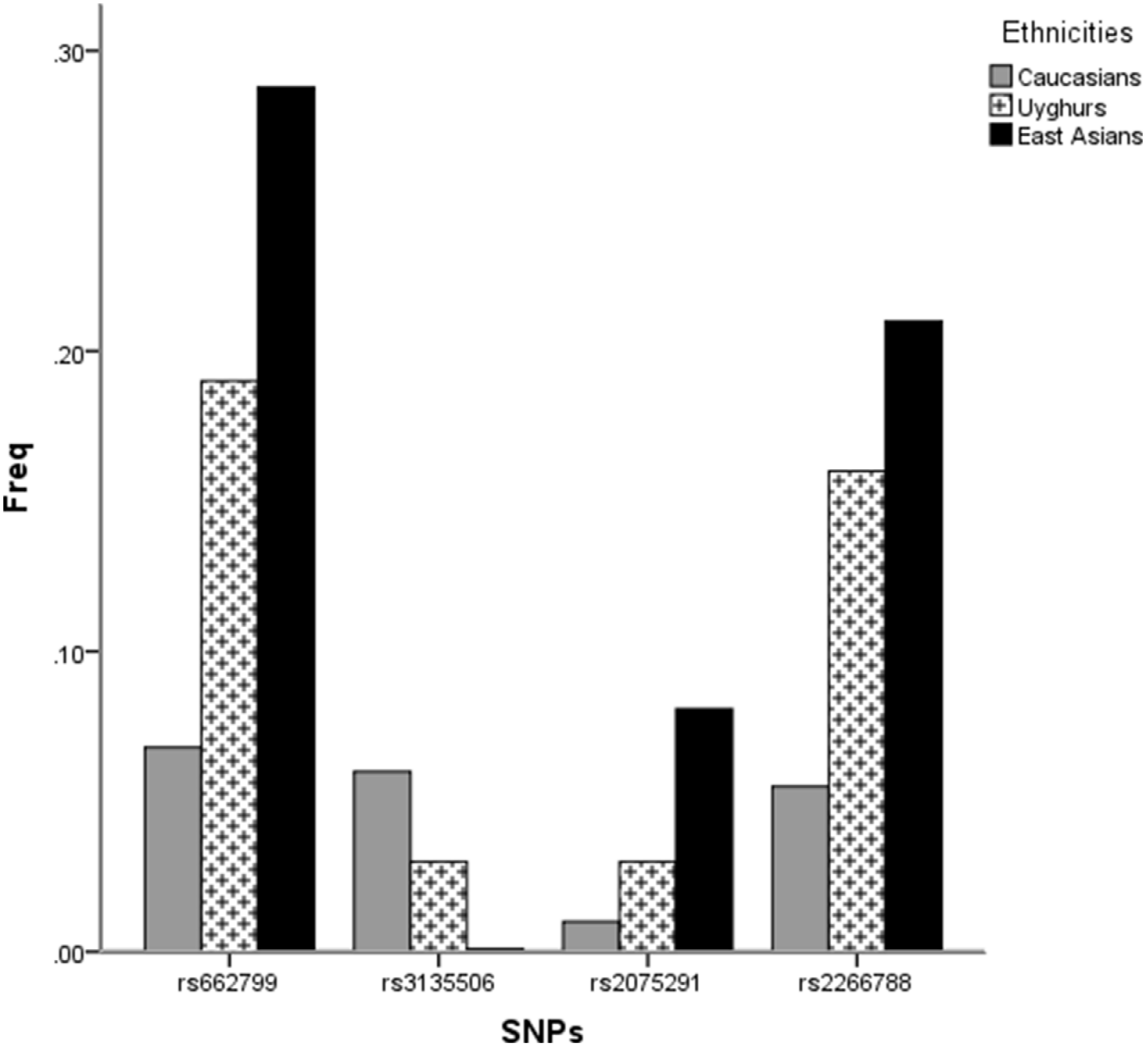
The minor allele frequencies (MAFs) of the polymorphisms in APOA5 in the different populations. Based on the online databases and literatures in addition to our own data.

**Figure 3:**
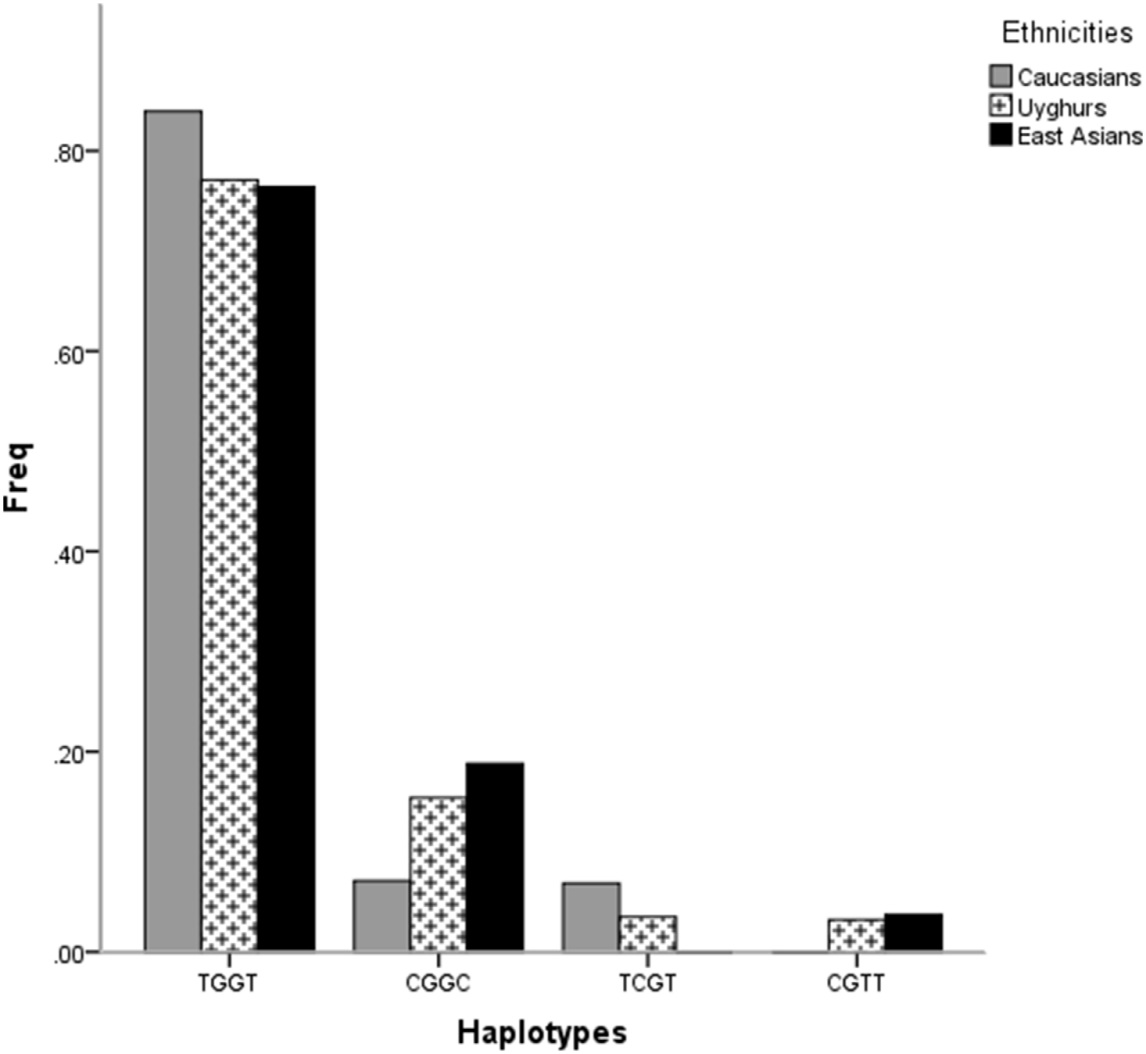
The frequencies of the haplotypes derived from the four SNPs in APOA5 in different populations. Based on the online databases and literatures in addition to our own data.

To compare the frequencies of the studied SNPs and haplotypes in Uyghurs to those in Caucasians and in East Asians, we searched the online databases and a total of 67 eligible studies were identified, including 20 studies involving Caucasians and 47 with East Asians (data not shown). Over half of them described the SNP rs662799, involving 10 reports on Caucasians and 33 on East Asians. However, studies involving the SNP rs3135506 in East Asians and rs2075291 in Caucasians were rare. Based on the data from these studies, in addition to the NCBI and Hapmap databases, the *APOA5* polymorphisms and haplotypes frequencies in different populations and the frequencies that were obtained in the general Uyghur population in the present study were demonstrated in Figures 2 and 3. The frequencies of the four SNPs in Uyghurs were in between those in Caucasians and in East Asians. The C allele of rs662799, C allele of rs2266788, and T allele of rs2075291 were more prevalent in Uyghurs compared with those in Caucasians and less prevalent compared to those in East Asians whereas the trend was opposite for the C allele of rs3135506 (all *P* for trend <0.05). The frequencies of the haplotypes conducted by the four SNPs in Uyghurs were also in between those in Caucasians and in East Asians although only marginal levels of significance were detected (*P* for trend ranging from 0.059 to 0.073).

### Association between *APOA5* gene SNPs/haplotypes and plasma TG levels in Uyghur population

The mean TG levels were compared with respect to the genotypes of the SNPs and haplotypes in the general Uyghur population to estimate the impact of the *APOA5* gene SNPs and haplotypes on TG levels. As shown in Table 2, the SNPs rs662799 and rs2266788 were significantly associated with TG levels at a multiple correction threshold level of *P*<0.0125 after controlling for age, sex, smoking and drinking. The other two SNPs, rs3135506 and rs2075291, were also associated with TG level, whereas the associations were not significant after multiple corrections. Compared with the most common haplotype TGGT, the haplotypes CGGC, TCGT, and CGTT were all significantly associated with TG levels (all adjusted *P*<0.0125, Table 3).

**Figure 4:**
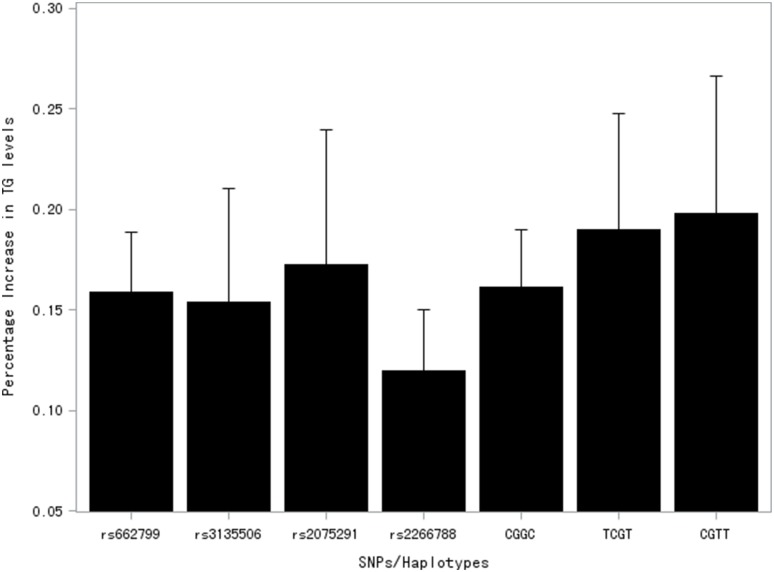
Mean effects and standard errors (bars) in TG levels for each SNP and haplotype.

Figure 4 shows the percentage increase of the TG means between the TG-raising minor allele carriers and the homozygotes of the normal allele for all four SNPs and also between the TG-raising haplotypes and the most common TGGT haplotype. Compared with non-carriers, carriers of the TG-raising minor allele of the four SNPs had 16.0%, 15.1%, 17.1%, and 12.4% higher TG levels for the SNP rs662799, rs3135506, rs2075291, and rs2266788, respectively. The effects of the three haplotypes on TG were in the range of 16.1% to 19.8%, with the haplotype CGTT, which was defined by the rare allele rs662799 and rs2075291, exhibiting the strongest TG-raising effect. The proportions of variance in TG explained by individual SNPs were 2.5%, 0.3%, 0.4%, 1.9% for rs662799, rs3135506, rs2075291, and rs2266788, respectively. To evaluate the combined effect of the four SNPs, we estimated the proportions of variance explained by the calculated haplotypes. The haplotypes explained 3.0% of the variances in TG levels in this population. With respect to other metabolic traits (TC, Glu, BMI, waist, SBP, and DBP), no significant associations were found with the exception that the T allele of the SNP rs2075291 inferred higher waist circumferences in this population (Table S1).

## Discussion

In this study, we aimed to identify the frequencies of the SNPs and the haplotypes in the *APOA5* gene and evaluated the effect of them on the plasma TG levels in Uyghur population, which is an admixture population of Caucasians and East Asians. We found that the presence of the minor alleles at each of the four studied SNPs in addition to the haplotypes constructed by them were associated with increased TG levels. To the best of our knowledge, this is the first study to correlate *APOA5* polymorphisms and haplotypes with plasma TG levels in the Uyghur population.

The association profiles between the SNPs at the *APOA5* locus and TG levels in Uyghurs differed from those in Caucasians and East Asians. The *APOA5* rs2075291 was previously found to correlate strongly with TG levels in East Asians but not in Caucasians or Turks [16]–[18], [26]. In the present study, we showed that the TG levels were higher in Uyghurs with the GT genotype compared with those possessing the GG genotype. Additionally, numerous studies have found associations between rs3135506 and TG levels in Caucasians but not in East Asians [27], [28]. In this study, we found that C carrier of rs3135506 had 15.1% higher TG compared with non-carriers. Notably, the haplotype CGTT, which was rare and not associated with TG in Caucasians, was also associated with elevated TG levels in the studied population.

Different gene-gene or gene-environment interactions may account for some of these discrepancies in addition to the different MAFs of the SNPs among ethnicities. Significant trends were observed for the MAFs of the four SNPs in *APOA5* in Caucasians, Uyghurs and East Asians, and their frequencies in Uyghurs were in between those of Caucasians and East Asians. The MAF of rs662799, rs3135506, and rs2266788 ranged from 0.06 to 0.08 in Caucasians. However, the MAFs of rs662799 and rs2266788 increased to more than 0.20 and the MAF of rs3135506 decreased to less than 0.01. Additionally, the MAF of rs2075291 was less than 0.01 in Caucasians and increased to approximately 0.04 in East Asians. The reasons for these differences were not clear. As TG is a key energy source in humans, the TG-raising allele may play an important role in human evolution [29]. The TG-raising allele frequencies were not consistently high or low in any one population makes the TG levels were comparable in the world.

The LD patterns of the *APOA5* SNPs in Uyghurs also differed from those in Caucasians and East Asians, with the LD between rs662799 and rs2266788 was stronger than that in East Asians but weaker than that in Caucasians. The different LD patterns in addition to the MAFs of the SNPs in *APOA5* led to the different haplotype profiles in this population. We found that the frequency of the haplotype CGGC (APOA5*2), which was defined by the rare alleles rs662799 and rs2266788, was 15.4% and was associated with 16.1% higher TG levels in Uyghurs. These results are consistent with those that have been reported in Caucasians and East Asians [7], [30], [31], indicating that the association between this haplotype and TG levels is irrespective of ethnicities. Meanwhile, we found that the haplotype CGTT (APOA5*4), which was defined by the rare alleles of rs662799 and rs2075291 with a frequency of 3.2%, was also associated with 19.8% higher TG levels. Our results are in concordance with previous studies demonstrating that the haplotype APOA5*4 is associated with 16% higher TG levels in Taiwan Chinese [15] and with familial combined hyperlipidemia in Hong Kong Chinese [14]. However, this haplotype had not been detected in Caucasians. The frequency of the haplotype TCGT (APOA5*3), which was defined by the rare allele of rs3135506, was similar to that of CGTT (APOA5*4) and was associated with 19.0% of higher TG levels in our population. The associations can also be observed in Caucasians but not in East Asians [32], [33].

The functions of the SNPs and haplotypes remain to be elucidated. The SNP rs3135506 was found in the second exon of the gene. The minor allele of this SNP was observed to lead to the vitiated signal activity of *APOA5* through amino acid replacement lowering the amount of functioning protein [34]. The SNPs, rs662799 and rs2266788, belonged to the well-defined haplotype APOA5*2, which was characterized by the rare allele of rs662799, rs651821, rs2072560, and rs2266788. Tight LD can be observed between the SNPs rs662799 and rs651821, and also between the SNPs rs2266788 and rs2072560 in both Caucasians and East Asians, suggesting that the haplotypes of these four SNPs can be inferred if we simultaneously genotyped two SNPs of them, one of rs662799/rs651821 and the other of rs2072560/rs2266788, similar to what was carried out in the present study. The well-documented hypertriglyceridemic effects of APOA5*2 were found to be partially due to the miR-binding site created by the rare allele of rs2266788 which mediated the *APOA5*-induced posttranscriptional downregulation [35]. However, in our study, we found that the haplotype CGTT (APOA5*4), defined by the rare allele of rs662799 and rs2075291, was also associated with TG, indicating that the SNP rs662799, together with rs2075291, may also have relevant biological functions. Although many studies aimed to demonstrate the function of the SNP rs662799 had been carried out, its functional effects remain to be elucidated. Since rs662799 is located within the promoter region of the APOA5 gene, it may affect mRNA translation, which could lead to lower plasma apoA-V levels, thus affecting plasma lipid concentrations. Indeed, some studies have found that subjects who are homozygous for the major allele of rs662799 have significantly higher plasma apoA-V levels compared with those who are homozygous for the minor allele [36]. However, the underlying mechanism leading to these occurrences is unknown. Meanwhile, few studies had been demonstrated the function of the SNP rs2075291. We performed bioinformatic studies to determine the effect of this SNP and found that the replacement of glycine with cysteine at amino acid 185 caused by this polymorphism were damaging (the SIFT and PolyPhen scores were 0.01 and 0.94, respectively). The TG-raising effects of the haplotype CGTT (APOA5*4) were induced by the SNP rs662799 or rs2075291 alone or by the combination of them needed to be studied further.

In conclusion, our cross-sectional study revealed the essential roles of the polymorphisms and haplotypes of *APOA5* in the dysregulations of TG levels in Uyghur population, which is an admixture population of Caucasians and East Asians. The functions of these SNPs and haplotypes require more extensive investigations in the future.

## Supporting Information

Table S1 Clinical characteristics according to the studied SNPs in APOA5 in the general Uyghur population.
(DOCX)
