# Supplementary material for: Influences of APOA5 Variants on Plasma Triglyceride Levels in Uyghur Population
Source: PLoS One. 2014 Oct 14;9(10):e110258. doi: 10.1371/journal.pone.0110258 (PMC4196964; doi:10.1371/journal.pone.0110258)
Supplement: Table S1 — Clinical characteristics according to the studied SNPs in APOA5 in the general Uyghur population. (DOCX) [file pone.0110258.s001.docx]

**Table S1** Clinical characteristics according to the studied SNPs in APOA5 in the general Uyghur population

|  | rs662799 | | |  | rs3135506 | |  | rs2075291 | |  | rs2266788 | | |
| --- | --- | --- | --- | --- | --- | --- | --- | --- | --- | --- | --- | --- | --- |
|  | TT | CT | CC |  | GG | GC/CC |  | GG | GT |  | TT | CT | CC |
| BMI (Kg/m^2^) | 26.7±4.65 | 26.5±4.45 | 26.2±4.17 |  | 26.6±4.57 | 26.9±4.61 |  | 26.6±4.60 | 26.5±4.14 |  | 26.7±4.63 | 26.3±4.44 | 26.5±3.83 |
| Waist (cm) | 88.3±11.7 | 88.3±10.9 | 88.3±9.48 |  | 88.3±11.4 | 88.6±11.5 |  | 88.1±11.4 | 91.2±10.4^$^ |  | 88.7±11.6 | 87.4±10.7 | 88.5±9.99 |
| TC (mmol/L) | 4.52±1.04 | 4.54±1.01 | 4.92±1.23 |  | 4.53±1.05 | 4.69±0.98 |  | 4.54±1.04 | 4.66±1.06 |  | 4.54±1.05 | 4.54±1.01 | 4.84±1.22 |
| Glu (mmol/L) ^*^ | 5.74±1.68 | 5.71±1.84 | 5.77±1.86 |  | 5.74±1.78 | 5.72±1.88 |  | 5.74±1.81 | 5.67±1.05 |  | 5.77±1.80 | 5.69±1.85 | 5.51±0.73 |
| SBP (mm Hg) ^#^ | 137.8±28.3 | 137.8±27.1 | 134.5±23.8 |  | 137.7±27.4 | 137.3±31.9 |  | 137.4±27.7 | 140.3±29.2 |  | 137.9±28.3 | 137.6±27.3 | 133.6±21.8 |
| DBP (mm Hg) ^#^ | 83.7±15.0 | 84.2±15.0 | 83.0±13.5 |  | 83.8±14.9 | 84.7±15.5 |  | 83.7±14.9 | 84.9±15.7 |  | 83.7±15.0 | 83.8±15.0 | 83.8±14.1 |

^$^ *P* <0.05 compared with the GG group.

^*^ only individuals who did not take antidiabetic drug were included.

^#^ only individuals who did not take antihypertensive drug were included.
